# Experimental Biomechanical Analysis of the Bone-to-Implant Connection in Single-Piece Implants

**DOI:** 10.3390/jfb16100393

**Published:** 2025-10-19

**Authors:** Karina Krawiec, Adam Kurzawa, Jakub J. Słowiński, Calin Romulus Fodor, Łukasz Pałka

**Affiliations:** 1 Department of Mechanics, Materials and Biomedical Engineering, Faculty of Mechanical Engineering, Wroclaw University of Science and Technology, Smoluchowskiego 25, 50-372 Wroclaw, Poland; karina.krawiec@pwr.edu.pl; 2 Department of Lightweight Elements Engineering, Foundry and Automation, Faculty of Mechanical Engineering, Wroclaw University of Science and Technology, Smoluchowskiego 25, 50-372 Wroclaw, Poland; adam.kurzawa@pwr.edu.pl; 3 Independent Researcher, 405300 Gherla, Romania; quick_dent@yahoo.com; 4 Independent Researcher, Rzeszowska 2, 68-200 Zary, Poland

**Keywords:** mandible implant, pullout test, bovine rib, artificial bone

## Abstract

The mechanical properties of dental implants are critical for their durability. The purpose of this study was to determine the maximum force required to induce full pull-out of a titanium implant from the bone and to characterize the mechanical behavior during this process. First, pull-out tests were performed on monolithic implants embedded in bovine ribs and foam blocks that mimic the mechanical parameters of human bone, allowing a quantitative evaluation of implant–bone interface strength and a comparison of geometric variants. Second, the extraction process was recreated in a three dimensional finite element model incorporating nonlinear interface contact and parameterization, enabling the reproduction of load–displacement curves; the results obtained showed good agreement with the experiment. Third, the fracture surfaces were observed macroscopically and by scanning electron microscopy/energy dispersive spectroscopy. The results demonstrated significant distinctions in the forces required to extract implants with varying thread geometries, clearly indicating the impact of implant design on their mechanical stability. The presented FEM-based methodology provides a reliable tool to study mechanical interactions at the implant–bone interface. The findings obtained can improve our understanding of implant behavior in biological systems and provide a basis for further optimization of their design.

## 1. Introduction

One of the key criteria in determining a patient’s suitability for implant therapy is the amount of bone available; specifically, the width and height of the alveolar ridge, and its biomechanical integrity [1,2,3,4]. Bone density serves as a surrogate for this mechanical quality; it is strongly correlated with primary stability (e.g., insertion torque) and influences both the rate of osseointegration and the timing of loading [5].

However, many patients still lack the quantity or quality of bone sufficient to proceed with the conventional implant placement without augmentation. Cone beam computed tomography studies reveal marked regional differences in cortical thickness and trabecular density, the bone most compromised generally found in the posterior maxilla [6,7,8]. In such situations, short implants (≤6–7 mm) can be a predictable alternative to extensive augmentation, providing survival rates and marginal bone loss comparable to longer implants placed simultaneously with bone grafting when indicated appropriately [9].

Tooth loss initiates a cascade of anatomical changes that reduce bone density and mechanical strength, especially at posterior sites where low density D3-D4 bone and thinner cortical plates are more common [7,10]. In the maxilla, sinus pneumatization and ridge resorption diminish residual bone height, while in the mandible, the position of the inferior alveolar nerve canal limits implant length and trajectory [11,12,13,14]. Consequently, patients’ chewing ability, aesthetics, and social relationships may be affected; cases with limited bone availability or poor bone quality are at increased risk of mechanical and biological complications that can lead to implant failure or loss and require prosthetic reconstructions [15,16,17,18].

In this context, this work combines experimental and numerical components designed for critical loading scenarios and realistic boundary conditions. First, pull-out tests were performed on monolithic implants embedded in bovine ribs and foam blocks that mimic the mechanical parameters of human bone, allowing a quantitative evaluation of implant–bone interface strength and a comparison of geometric variants. Second, microscopic analysis of the implants showed the presence of fatigue crack initiation zones in the implant neck area, which may be the result of cyclic loading and stresses generated during implant adjustment during surgical procedures. Third, the extraction process was recreated in a three-dimensional FE model incorporating nonlinear interface contact and parameterization, allowing the reproduction of load–displacement curves; the results obtained showed good agreement with the experiment.

## 2. Materials and Methods

Single part implants with different shapes were compared. Each implant was inserted into a known density polyurethane (PU) foam, for example, in the bone of the ribs of bovine (purchased from a local butcher shop). The pull-out test was performed under controlled mechanical and environmental conditions, recording maximum pull-out force and damage patterns. The experimental setup of the biological material was reproduced by finite element analysis (FEA), corresponding to implant geometry, material properties, boundaries, and contact conditions. The fracture surfaces were observed macroscopically and by SEM/EDS to characterize the failure modes and materials composition. The numerical models were validated against the experimental force–displacement data and showed high quantitative consistency.

### 2.1. Implants and Substrates

**Figure 1:**
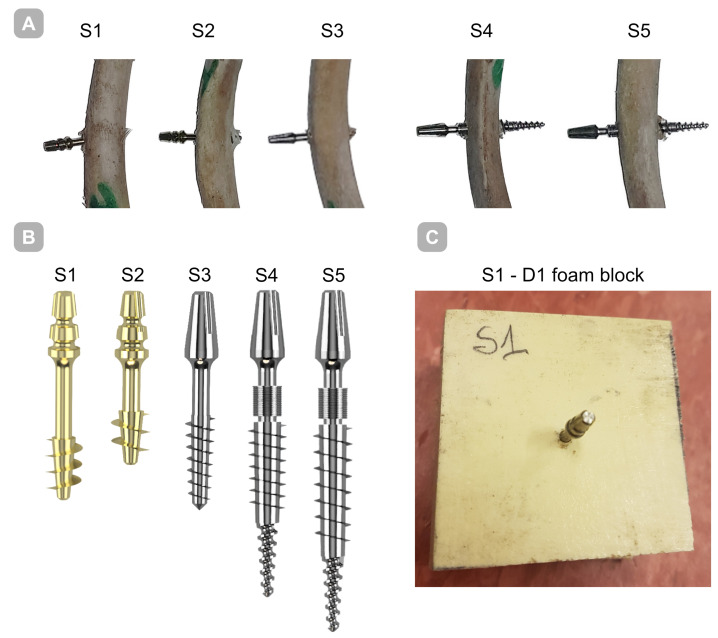
Implants employed in the present investigation. (**A**) Implant embedded in bone; (**B**) implants shown together on a single scale to highlight dimensional differences; (**C**) implant embedded within a polyurethane foam block.

Five titanium (Ti-6Al-4V) implants (BCS, Ihde-Dental GMBH, Munich, Germany) S1–S5 with distinct thread geometries were evaluated—Figure 1. Implants differed in working thread dimensions and guide tip design. Substrates were bovine rib bone specimens prepared to preserve hydration and rigid polyurethane (PU) foam blocks (50 × 50 × 50 mm; Promedicus, Mikołów, Poland; cat. no. 10-9010/10-9015) representing densities D1–D4 per ASTM F1839-08 [19] surrogate classification; block mechanical properties are summarized in Table 1. With densities D1–D4, we refer to four nominal levels of apparent density of the blocks used to simulate spongy bone with rigid polyurethane foam, where D1 is the densest/hardest material and D4 is the rarest/loosest, according to the Misch classification [20].

### 2.2. Specimen Conditioning and Site Preparation

The bones were stored at −20 °C to prevent biological degradation and loss of mechanical properties. Prior to testing, the specimens were thawed at room temperature for 2 h. During thawing they were wrapped in gauze saturated with a 0.9% NaCl solution, after which they were rehydrated with saline to restore their native hydration state.

All tests were conducted at room temperature, which is approximately (22 ± 2 °C) and 50±5% relative humidity. The process of preparing implant sites in animal bones and polyurethane foam was carried out using an incremental osteotomy protocol in which successive drills of increasing diameter were employed to progressively enlarge the cavity while preserving cortical bone integrity. This technique was chosen because it is known to enhance the primary stability of implants. Specialized instrumentation, including step drills and a torque wrench for controlled tightening of the implants, was used.

**Figure 2:**
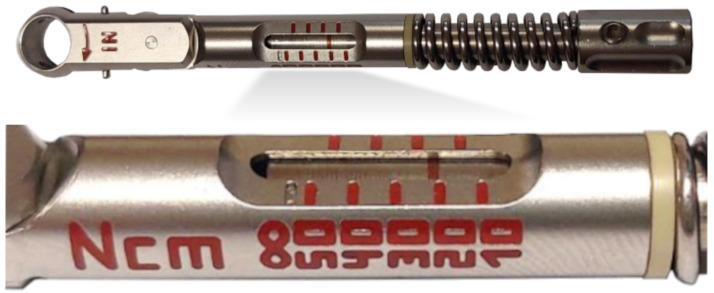
MDI torque wrench with indicator (Torque wrench, B&B DENTAL Implant Company, San Pietro in Casale, Italy).

Each implant site was prepared in an identical manner, ensuring uniform conditions for the experiment and allowing comparability of the results obtained. The procedure included the following steps:

- Pre-drilling using a drill with a 1.0 mm diameter.
- Final preparation of the implant site, according to the implant manufacturer’s guidelines.
- Tightening the implant with a torque wrench, taking into account the specified force torque. The tool used is presented in Figure 2.

### 2.3. Mechanical Testing (Pull-Out)

Mechanical tests were performed on a universal testing machine (MTS Systems, Eden Prairie, MN, USA) for the uniaxial tensile test. A constant displacement of 0.1 mm/s was given. The first part of the performed experiments focused on evaluating the pull-out force of the implants (designated S1–S5) through the test of pulling them out of bovine rib bones. The pull-out process continued until the implant was completely removed from the bone, which corresponded to the situation when the force value dropped to zero and the implant completely lost contact with the bone substrate. The purpose of this research was to determine the maximum force required to induce full pull-out of the implant from the bone and to characterize the mechanical behavior during this process. During the pull-out test, the displacement values corresponding to the applied forces were recorded using a dynamometer.

In the second stage, tests were performed on rigid foam blocks. For this purpose, pull-out tests were performed for each specimen D1–D4 for implant S1 and polyurethane foam D4 for implants S1 to S5 at a constant speed.

Each implant was prepared and assembled in a standardized manner, allowing precise analysis of the effect of implant conditions on implant stability. By using the described procedure, it was possible to obtain consistent and reliable test results.

**Figure 3:**
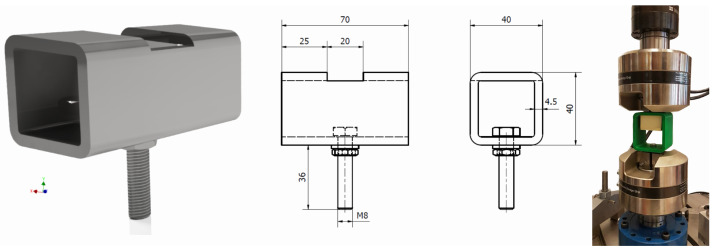
Sample holders used in experiments.

In order to ensure clean extraction characteristics, a holder had to be made to provide support to the upper part of the samples. For this purpose, a mounting for samples was made of a square profile presented in Figure 3; the profile was made of Steel Standard EN C50 [21] with a hardness above 56 HRC, Rm = 650.0 MPa, Re = 355.0 MPa, A = 13%, ensuring adequate stiffness of the system.

A similar holder, fabricated from PETG (material properties in Table 2) using the 3D printing (FDM) method on an Original Prusa MK4 printer (Prusa Research, Prague, Czech Republic), was prepared for the polyurethane specimens. The holder dimensions were selected to provide approximately 5 mm of clearance for the specimen during mounting, and the wall thickness was set to 5 mm—Figure 3; far-right position.

### 2.4. Numerical Analysis

**Figure 4:**
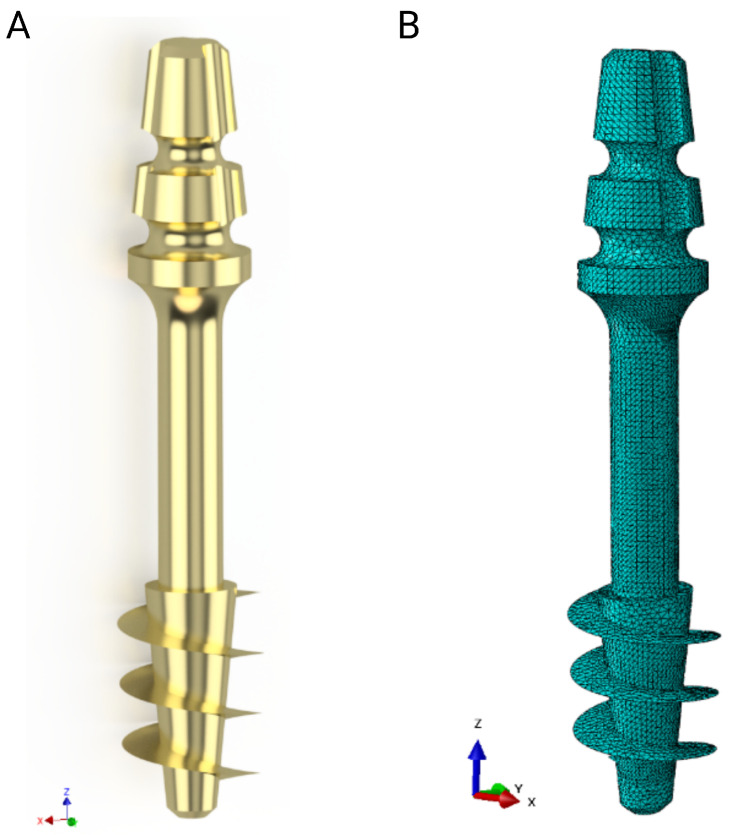
S1 implant: (**A**) Geometrical model, (**B**) numerical model.

Strength calculations of the bone–implant contact were carried out in Ansys Workbench 2024 R2 (Ansys Inc., Canonsburg, PA, USA). For this purpose, geometric models of the implants were made in Inventor, which were then exported in step format to the computational environment. A detailed geometric model was prepared for each of the dental implant models, taking into account the parameters of the threads, which made it possible to accurately represent the actual mechanical properties of the implants. Figure 4 shows an example image of the geometric model of the S1 implant and its numerical model after discretization.

The mathematical model of Johnson–Cook (J-C) was used for elastic–plastic materials of titanium alloy, taking into account the plastic part.

(1) $$\sigma_{y}=\left(A+B{\bar{\varepsilon }}^{p^{n}}\right)\left(1+c\ln{\dot{\varepsilon }}^{*}\right)\left(1-T^{*m}\right)$$

(2) $${\dot{\varepsilon }}^{*}=\frac{{\dot{\bar{\varepsilon }}}^{p}}{{\dot{\bar{\varepsilon }}}_{0}}$$

(3) $$T^{*}=\frac{T-T_{\mathrm{room}}}{T_{\mathrm{melt}}-T_{\mathrm{room}}}$$

in which

*A*—initial yield strength of the material, *B*—strain hardening coefficient, *n*—strain hardening exponent, *c*—strain rate effect, *m*—thermal softening effect, ${\bar{\varepsilon }}^{p^{n}}$—the equivalent plastic strain, ${\dot{\bar{\varepsilon }}}^{p}$—the plastic strain rate, ${\dot{\varepsilon }}^{*}$—dimensionless effective strain rate, ${\dot{\bar{\varepsilon }}}_{0}$—reference strain rate, $T^{*}$—normalized temperature, $T_{\mathrm{room}}$—reference temperature (room), $T_{\mathrm{melt}}$—melting temperature, and *T*—base temperature (present).

The part related to the strain rate and thermal weakening was omitted due to the quasi-static nature of the phenomenon. The mechanical properties of the Ti-6Al-4V alloy, the cortical bone, and the trabecular bone used in the numerical analysis are shown in Table 3, Table 4 and Table 5.

**Figure 5:**
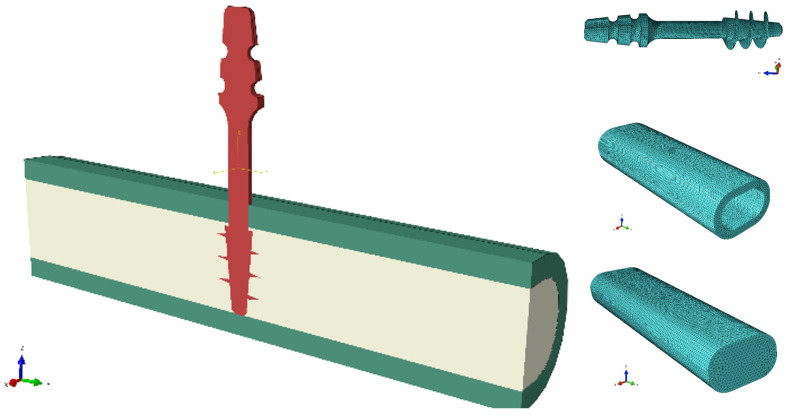
An exemplary combination of a geometric model and components of numerical models.

The explicit method with an integration step of 0.001 s was used for the calculations. High-order 10-node tetrahedral elements with an average size of 0.2 mm were used for all models. This type of element is suitable for the modeling of irregular objects, such as implant threads in this case. At the same time, the selected element size offers a reasonable compromise between the accuracy of the result distribution and the computation time. Further reduction of elements does not lead to significant changes in the distribution of analyzed quantities, but the computation time is unacceptable. Body interactions between the implant and the bone were defined as frictional contact, with friction coefficients of 0.38 for static friction and 0.3 for dynamic friction [24,25]. The connection between compact and trabecular bone tissue has been defined as bonded contact. Figure 5 shows the geometric model of the assembly used in the simulations and the individual component discretizations.

### 2.5. Fracture Analysis

The next stage of our research focused on material analysis to assess possible failure mechanisms that may occur in clinical practice. The neck region of the implants, a critical region where the smallest cross-sectional diameter exists between the threaded body and the abutment, was particularly interesting. According to manufacturer specifications, these implants are designed with a maximum insert torque of 80 Ncm; exceeding this threshold may lead to structural failures, especially in the neck due to stress concentrations [26,27]. Our analysis aimed specifically to determine whether material properties contribute to this type of damage and to identify strategies to improve the durability of implants and prevent similar failures in vivo.

The Hitachi TM3000 scanning electron microscope (Angstrom Scientific, Inc., Tokyo, Japan) integrated with the Energy Dispersive Spectroscopy (EDS) system (EDAX, Pleasanton, CA, USA) was used to analyze the chemical and phase composition of the samples. The chemical composition is presented in Table 6. The content of the alloying elements is within the range required by the standard guidelines for the Ti-6Al-4V alloy [28].

Structural tests were performed on metallographic microsections made on longitudinal sections of samples subjected to etching with Kroll’s reagent. Observations were made with the use of a NIKON Eclipse MA200 light microscope (Eclipse MA200, Nikon Corporation, Tokyo, Japan).

## 3. Results

### 3.1. Pull-Out in Bovine Rib Bone

**Figure 6:**
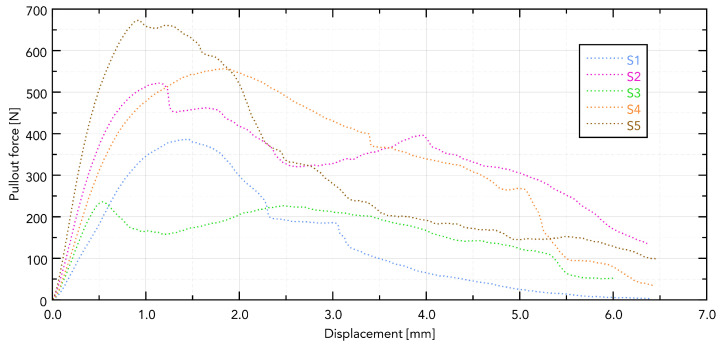
Displacement forces for individual samples.

The force–displacement curves for implants S1–S5 extracted from bovine rib bone showed distinct profiles with a single dominant peak followed by a rapid force drop, consistent with a loss of thread engagement (Figure 6).

Visible differences in the force needed to pull out the implant are directly related to its shape. The greatest force was observed in the S5 implant, which can be directly translated into the length of the working part of the implant and the size of the working thread. The guiding part in samples S4 and S5 does not affect the force needed to pull them out.

**Figure 7:**
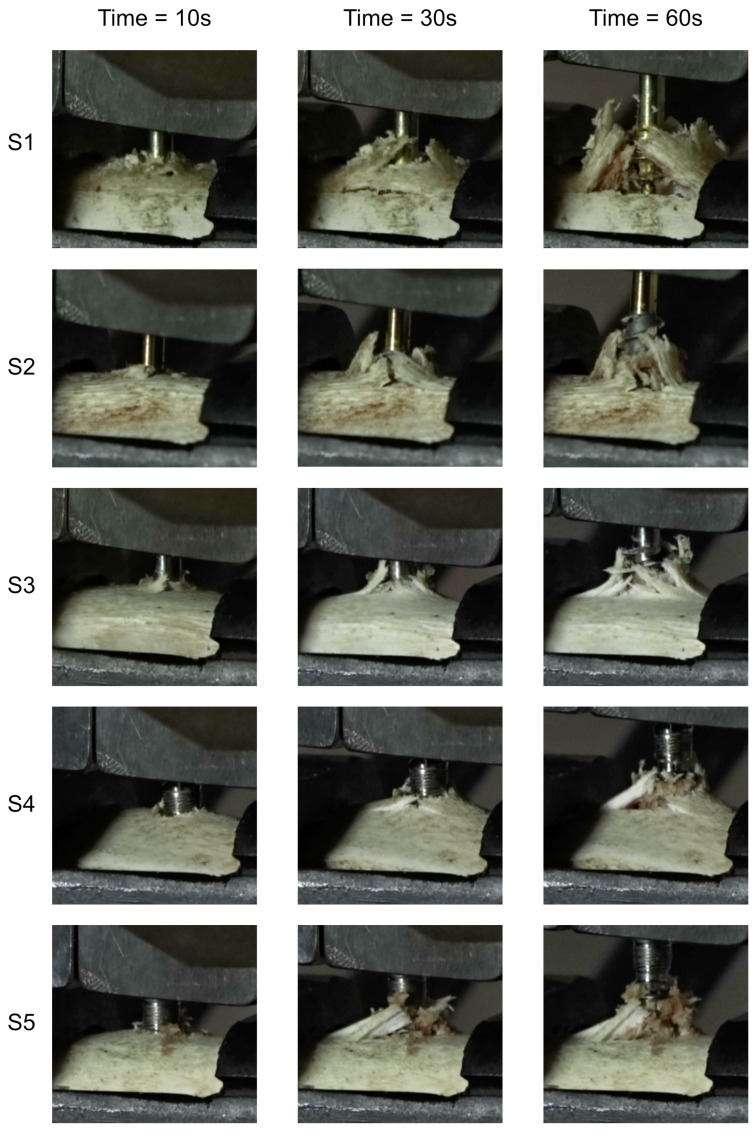
Temporal progression of bone tissue damage in defined time intervals during the implant extraction process.

The damage to the samples during the tests is presented in Figure 7. External destruction of the bone (cortical) and tearing of the internal part (trabecular) are visible. The greatest destruction was observed in the samples with the geometrically widest working part of the thread, which directly translates into the greatest force needed to pull out the implant.

### 3.2. Pull-Out in PU Surrogates, Density Series (D1–D4)

The destruction mechanism observed after the pull-out test followed a distinct cylindrical plane pattern, which was formed as a result of the material shearing along the surface defined by the radius of the outer edge of the thread. This specific failure mode highlights the critical role played by the geometry of the implant’s threads in determining how mechanical forces are transmitted and resisted by the surrounding bone structure.

(4) $$\tau_{t}=\frac{F}{A}\leq k_{t}$$

(5) $$A=2\pi \frac{d}{2}\;H$$

where *F*—compressive force, *A*—shearing plane, *d*—diameter, *H*—thread height.

During the pull-out process, the implant threads, bonded with the bone material, created localized stress concentrations at the interface. As the applied force increased, these stresses exceeded the shear strength of the bone-like blocks, leading to a progressive failure that manifested itself as the cylindrical shearing plane.

**Figure 8:**
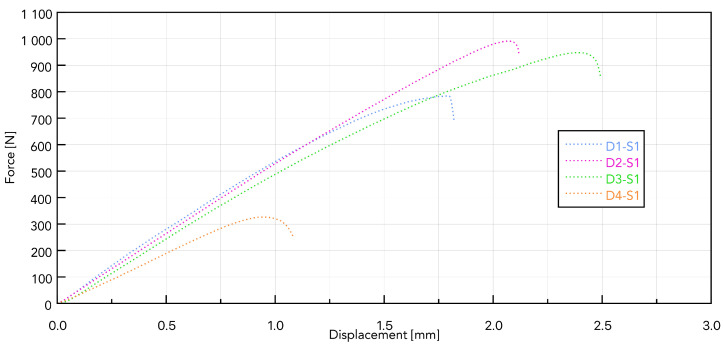
Force–displacement curves for samples D1 to D4 based on the S1 implant.

Presented in Figure 8, the force–displacement diagram for samples D1 to D4 with the S1 implant shows that the greatest force needed to remove the implant is for sample D2 and is 996.9 N (Table 7).

It can be assumed that, with a continuous pilot hole diameter throughout the density range, D1 (the hardest) may experience local compression during self-cutting, which reduces effective thread engagement and reduces load capacity during installation. In D2 (medium density), the spring-back effect provides better lateral pressure on the thread profile, a higher friction ratio, and a larger cutting surface, leading to a higher pulling force [29].

The first analysis of the S1 implant and all PU samples was carried out to select foam with a density as close as possible to the bone density of the biological samples. After determining that D4 foam is the most suitable, all analyzed implants (S1–S5) were pulled out.

**Figure 9:**
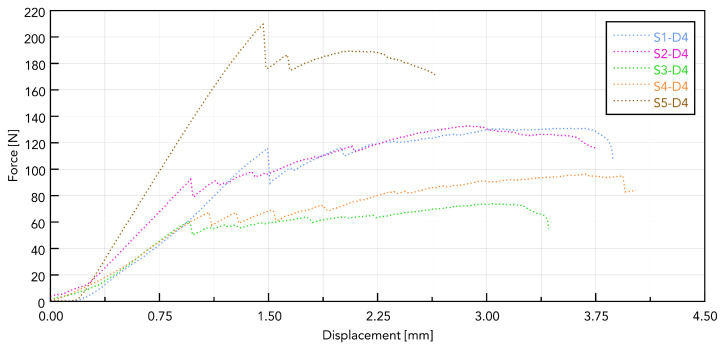
Force–displacement curves for samples S1 to S5 based on polyurethane foam.

Figure 9 shows a summary for one selected D4 polyurethane foam, which mimics bone structure. The test was conducted for pulling all five types of implants. The presented graph illustrates the force–displacement relationship during the pull-out test of various types of dental implants (labeled S1–S5) carried out using a PU bone block that simulates bone tissue (Promedicus, Mikołów, Poland; cat. no. 10-9010/10-9015). The curves represent different implant types, revealing variations in maximum pull-out force and displacement behavior, indicating differences in mechanical performance between the tested implants. The results demonstrate significant distinctions in force profiles, and some implants achieve higher maximum forces and display different stabilities under similar test conditions. The S5 implant reached the highest maximum force (210 N), characterized by a sharp increase in resistance and a sudden decrease after reaching the peak. The other implants (S1, S2, S3, and S4) generated lower maximum forces (60–140 N), showing a smoother destruction course. The pull-out force was closely related to the diameter, length, and number of thread turns of the implant. A clear relationship was observed, indicating that the larger the diameter and the area of the threads, the greater the pull-out force. The pull-out properties of implants S1, S2, S3, and S4 were very similar, maintaining the above trend. The exceptionally high pull of the S5 implant can be associated with a larger implant area. It should be noted, however, that this study was pilot in nature and that the results obtained are considered to be exploratory.

Images of structures made with magnifications of 200× and 1000× are shown in Table 8. The structure in all samples tested is characterized by a band distribution of the β phase, arranged mainly longitudinally on the grain boundaries of the α phase. The band structure results from the applied manufacturing technique with accompanying significant plastic deformations.

In the Ti-6Al-4V alloy, aluminum acts as an α stabilizer by increasing the α-β transition temperature, and vanadium acts as a β stabilizer. The EDS analysis allowed one to specify the chemical composition of the α and β phases. The α phase was found to be richer in aluminum, while the β phase is richer in vanadium. Table 9, for the example of sample S4, shows how to determine the chemical composition—Table 10.

**Figure 10:**
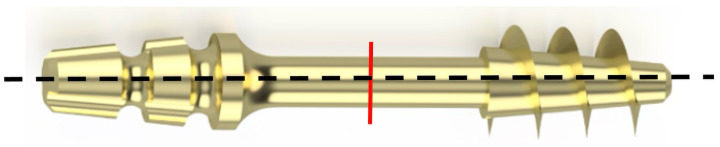
The red line indicates the place on the implant neck subjected to fracture surface analysis.

To determine the nature of the fracture and analyze the surface of the fracture, the implant was subjected to transverse bending—Figure 10. For each sample, five bends were performed at an angle of up to 30° relative to the longitudinal axis until the sample fractured.

**Figure 11:**
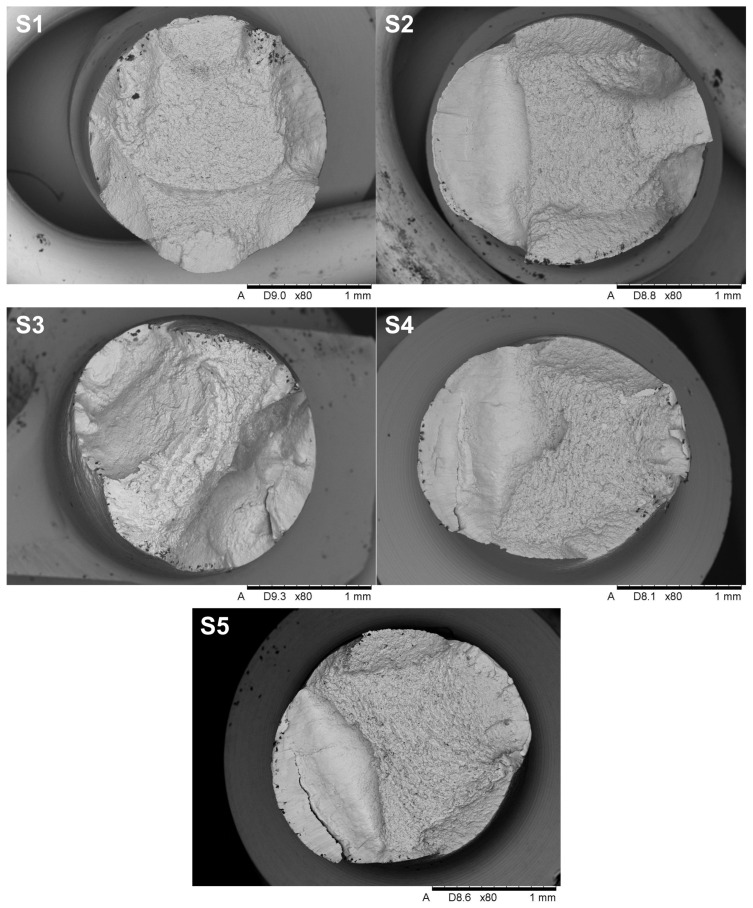
The overall view of the fracture surfaces.

SEM images of the individual implant fracture on the transverse surface in the cervical section are presented in Figure 11, showing a typical visible fracture pattern with an initial crack caused by bending the neck of the implant. The observed fractures are characteristic of the generation of cyclic stress within the structure, particularly in the cross-sectional area of the implant. These stresses were induced by bending the implant neck at an angle of up to 30° relative to the longitudinal axis. This simulated the positioning of the implant during a surgical procedure. Each implant test demonstrated durability for a maximum of five such deflections, after which fracture occurred.

The fractures in implants S2, S4, and S5 exhibit characteristic features of fatigue and brittle failure, consistent with the mechanisms described in the literature on crack propagation. On the left side of the cross-section, a smooth surface is visible, typical of fatigue fractures that develop gradually under cyclic stresses. The smoothness of this surface is the result of friction between the contact surfaces during cyclic loading, which confirms the mechanism of propagation of fatigue cracks. On the right side of the cross-section, the characteristic of brittle fractures dominate, characterized by a rough and irregular surface, indicating sudden material failure after critical stress levels are reached. This transition from fatigue to brittle fracture is typical for materials subjected to variable loads, where progressive weakening of the structure ultimately leads to abrupt failure. Additionally, oval-shaped indentations are observed along the edges of the cross-section, providing clear evidence of the bending direction during implant loading. The analysis of these characteristics, in the context of microstructure and fracture mechanics, suggests that failure occurred in two stages: initial crack growth of fatigue in the area of maximum bending stress, followed by sudden brittle fracture when the material reached its critical strength. These mechanisms align with fatigue and brittle crack propagation models widely documented in the engineering literature. Samples S1 and S3 do not exhibit a fatigue zone, but instead display solely brittle fracture characteristics, which may indicate a failure mechanism dominated by an overload condition rather than progressive damage. The absence of a smooth fatigue surface and the prevalence of a rough, irregular fracture plane suggest that these samples failed abruptly under a single loading event that exceeded the material’s critical tensile or shear strength. This type of fracture behavior can be attributed to a lack of sufficient plastic deformation capacity in the material or the presence of stress concentrators, such as microstructural defects or manufacturing imperfections, which reduced overall structural integrity. Brittle fractures are often associated with high loading rates, low temperatures, or specific material properties, such as high stiffness and low toughness, which prevent the material from redistributing stresses before failure. The morphology of the fracture surface in samples S1 and S3 further supports the hypothesis that these fractures occurred due to the rapid propagation of cracks initiated in regions of stress concentration, without the gradual crack growth characteristic of fatigue.

### 3.3. Numerical Analysis

**Figure 12:**
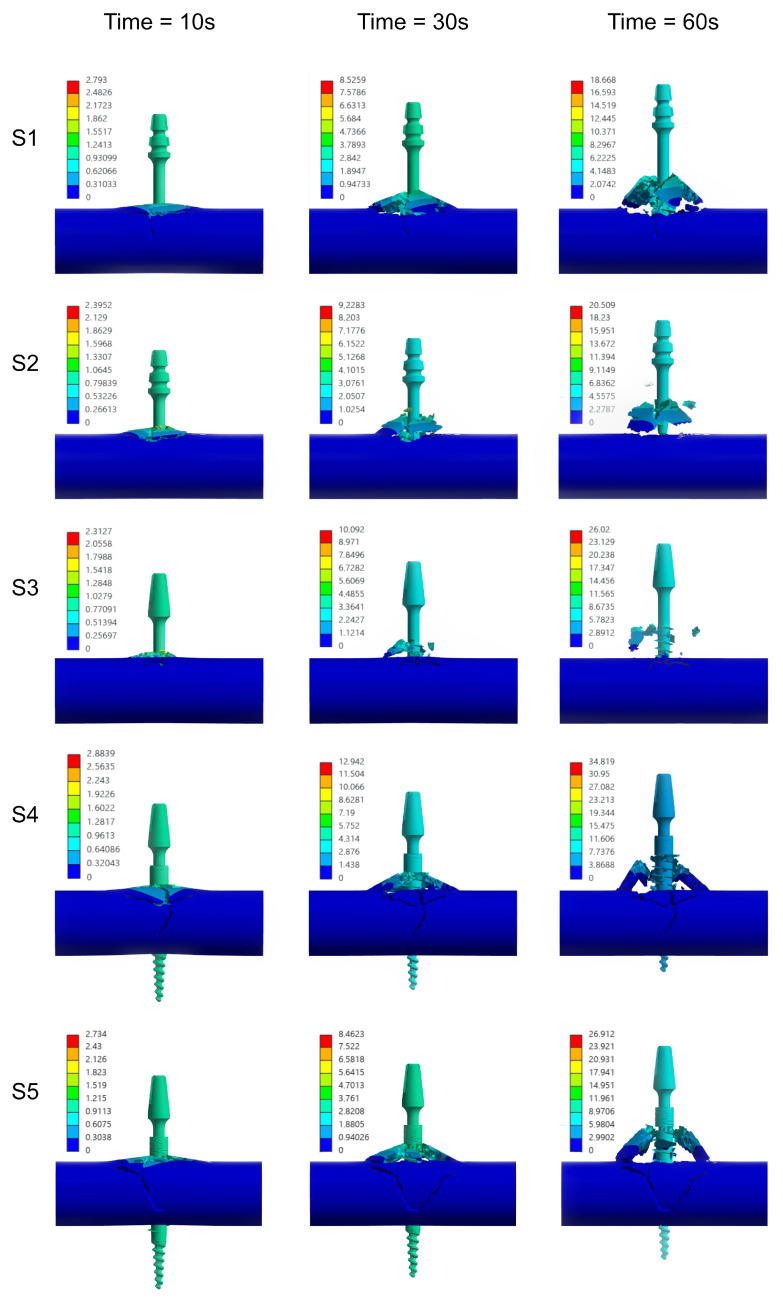
Temporal progression of bone tissue damage in defined time intervals during the implant extraction process—numerical analysis.

**Figure 13:**
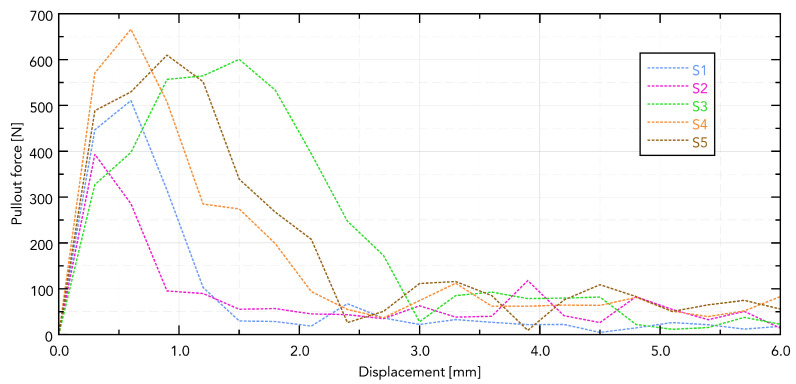
Force–displacement curves for samples—results from calculations.

Within the numerical analyses performed, the nodal displacement distributions were obtained and a change in reaction force was recorded as a consequence of implant removal—Figure 12. The computed results are in good agreement with the presented experimental data regarding the magnitude of the peak values. However, it should be noted that the temporal evolution of the reaction force curves deviates from the trends observed experimentally—Figure 13. This discrepancy arises from the simplified geometric representation and the damage model used in the simulations.

The experimental values and those obtained in the calculations were compared-Table 11, which allowed us to determine the relative error with the sign according to the formula.

(6) $$\varepsilon_{\%}=\frac{F_{\mathrm{calc}}-F_{\exp}}{F_{\exp}}\times 100\%$$

in which $\varepsilon_{\%}$—signed relative error, $F_{\mathrm{calc}}$—force obtained from calculations, and $F_{\exp}$—force measured experimentally.

The presented numerical model demonstrates good agreement with the experimental values of maximum pull-out force. However, for sample S3, the observed deviation was significantly greater, largely due to the heterogeneity of the biological material; the bones obtained may have had different microstructures and mechanical parameters despite due care.

## 4. Discussion

Dental implants have seen a significant increase in popularity in recent years. In the United States, the proportion of people with dental implants increased from 0.7% in 1999–2000 to 5.7% in 2015–2016. Further growth projections for 2026 range from 5.7% in a pessimistic scenario to as much as 23% in an optimistic scenario [30]. The increasing prevalence of edentulism, as well as the increasing number of people seeking effective tooth replacement options, are the main factors that contribute to this growth. Dental implants are increasingly regarded as a durable, functional, and aesthetic solution that outperforms traditional dentures in terms of stability and longevity.

However, as Wang et al. [31] point out, implant perceptions can be misleading. Many individuals exposed to varying-quality information about implants consider them a “panacea” for tooth loss, often overestimating their functionality and durability. In contrast, the high costs and invasive nature of implant procedures, which carry risks of complications and potential failure, can discourage patients. Despite these concerns, dental implants have a documented 10-year survival rate that exceeds 90% [32]. However, Alghamdi et al. [33] argue that this success may be challenged in the future by the aging population, where an increasing number of patients present with comorbidities such as diabetes, osteoporosis, drug-induced bone loss, and obesity. These conditions may adversely affect the osseointegration process, increasing the risk of implant failure.

Studies on implant failures show two time profiles. The long-term biological complications associated with peri-implantitis are visible, while technical problems and complications (veneer fractures, implants fractures) are visible shortly after implantation (or even during implantation) [34,35,36,37]. The nature of these fractures is strongly associated with the duration of use, with metal fatigue identified as the dominant failure mechanism. In implants made from titanium alloy Ti-6Al-4V, fatigue fractures can occur even at relatively low cyclic load levels [38].

The results of this study confirm these observations, indicating that fractures in Ti-6Al-4V implants are predominantly fatigue-induced, and cracks generally begin at a distinct starting point. Based on clinical experience, it is hypothesized that these initial cracks result from bending of the implant neck during dental prosthesis adjustment. Strietzel et al. [39] observed that bending the implant neck up to 30° during initial adjustments creates microcracks approximately 5 microns wide and 100 microns long. These microcracks, combined with occlusal loads, can act as initiation points for fatigue fractures, ultimately leading to failure. Our study demonstrated that bending of the implant neck and associated microcracks are critical factors in implant durability, warranting further analysis of their impact on the neck surface during initial adjustments.

In this study, the mechanical properties of dental implant connections to bone were determined, focusing on pull-out strength and microcrack analysis initiated during implant adjustment. This research is particularly important for improving implant durability under clinical conditions and minimizing the risk of implant damage. A key component was the determination of reference values for the forces required to extract the implants, providing a better understanding of their behavior under various load conditions.

The methodology of the study included both experimental tests and numerical simulations. Pull-out tests were performed on Ti-6Al-4V titanium implants using bovine bone and blocks that mimicked the mechanical parameters of human bone. The numerical simulations were based on a FEM method, allowing an accurate representation of the bone structures. The experimental results demonstrated differences in the forces required to extract implants with varying thread geometries, clearly indicating the impact of implant design on their mechanical stability.

The study produced numerical models that closely approximate the behavior of a real implant under load. Despite the pilot nature of this research, it is reasonable to assume that the implant model developed by experimental validation could be used to simulate more complex problems (e.g., treatment planning).

## 5. Conclusions

Finite Element Method (FEM) enabled the development of a highly satisfactory model for the extraction of a titanium implant from bovine bone. The difference between the results of the numerical analysis and the reference values for the mechanical properties of the elements tested was less than 10% in most cases. A significant error of 60% was observed in only one sample, which we attribute to the incoherence (high heterogeneity) of bone material.

The accurate representation of the trabecular bone architecture in the numerical model was a key element in the precision of the simulation. The use of the Johnson–Cook material model allowed the capture of the effects of strain rate, temperature, and ultimate strain, providing a more accurate reconstruction of the mechanical response of bone tissue under loading conditions.

The observed discrepancies between the results of the numerical simulation and the experimental extraction tests are mainly due to the necessary simplifications of the model (including assumptions about geometry and boundary conditions) and the inherent variability of the bovine bone properties (inhomogeneity in density, composition, and microstructure), which influence the mechanical response.

In this study, the use of bone-imitating samples proved effective in replicating the mechanical properties of biological tissue, enabling the standardization of the test conditions while maintaining the adequacy of the in vivo behavior.

In conclusion, the presented FEM-based methodology supported by a detailed characterization of the material and a robust mechanical model provides a reliable tool to study mechanical interactions at the implant–bone interface. The results obtained can improve our understanding of implant behavior in biological systems and provide a basis for further optimization of their design. Future work should incorporate patient-specific data and systematically analyze the impact of bone heterogeneity on the accuracy of numerical predictions.

## 6. Limitations

The authors point out that some limitations of the study should be taken into account when finalizing the work.

- Small sample sizes (n = 5, pilot) limit the power of inference and increase the risk of I/II errors; therefore, no formal significance tests, comparisons between implant types, and statistical analyses have been carried out.
- The single source of bone material and the narrow density range (species/anatomical origin) limit generality; the possible influence of individual and anatomical differences has not been assessed.
- Sample preparation (freezing/steaming, watering, and exposure time/temperature) may have changed the mechanical properties of bones; humidity/temperature during the tests has not been fully controlled.
- Socket machining (drilling/remolding, tolerances, and axiality) and the lack of post-machining diameter and roughness measurements may have introduced seat variation that affects pull-out force.
- Mechanical protocols limited to quasi-static loading in one direction and speed–no fatigue/impact tests and multiaxial conditions, limiting the ecological validity of clinical conditions.
- Lack of 3D imaging (e.g., micro-CT) before and after the test—the actual shape of the socket, microcracks, and bone contact with the implant have not been verified.

In the main study, we plan to have the following: a larger sample group, precise control of hole formation and implant placement, micro-CT to assess damage and contact, fatigue tests, statistical analysis with multiple comparison corrections, and independent validation of models.

## Figures and Tables

**Table 1:** Characteristics of rigid polyurethane (PU) foam blocks.

No.	Hardness	Compressive Strength	Flexural Strength	Density
	[Shore D]	[MPa]	[MPa]	[g/cm^3^]
D1	76	34.0	30.0	0.700
D2	63	20.0	20.0	0.500
D3	47	10.0	8.0	0.245
D4	37	5.0	6.0	0.325

**Table 2:** Material properties of PETG holder [22].

Material	Manufacturer	Density	Hardness	Tensile Strength	Elasticity Modulus
		[g/cm^3^]	[Shore D]	[MPa]	[MPa]
PETG	TERVATIVE, Warsaw, Poland	1.26	70.0	84.6	970

**Table 3:** Mechanical parameters of the Ti-6Al-4V alloy.

Parameter	Value	Unit
Density (ρ)	4429.0	kg/m^3^
Young’s modulus (*E*)	111.2	GPa
Poisson’s ratio (ν)	0.34	-
Johnson–Cook Model		
Yield strength (*A*)	862.0	MPa
Fixed fortification (*B*)	331.0	MPa
Exponent of consolidation (*n*)	0.34	-

**Table 4:** Mechanical parameters of the cortical bone, reprinted from Ref. [23].

Parameter	Value	Unit
Density (ρ)	1850.0	kg/m^3^
Young’s modulus (*E*)	1.80	GPa
Poisson’s ratio (ν)	0.35	-
Johnson–Cook Model		
Yield strength (*A*)	160.0	MPa
Fixed fortification (*B*)	165.0	MPa
Exponent of consolidation (*n*)	0.1	-

**Table 5:** Mechanical parameters of the trabecular bone reprinted from Ref. [23].

Parameter	Value	Unit
Density (ρ)	900.0	kg/m^3^
Young’s modulus (*E*)	482.0	MPa
Poisson’s ratio (ν)	0.4	-
Johnson–Cook Model		
Yield strength (*A*)	42.0	MPa
Fixed fortification (*B*)	55.0	MPa
Exponent of consolidation (*n*)	0.9	-

**Table 6:** Chemical composition of samples S1–S5.

Sample	Amount [% Weight]		
	Ti	Al	V
S1	90.6	5.8	3.6
S2	90.7	5.8	3.5
S3	90.4	5.7	3.9
S4	90.7	5.6	3.7
S5	90.9	5.6	3.5

**Table 7:** Pull-out force needed to remove implant from polyurethane foam.

Sample	D1	D2	D3	D4
Max force [N]	793.3	996.9	969.4	332.2

**Table 8:** Images of the microstructure seen in optical and electron microscope.

Sample	Optical Microscope	Electron Microscope
S1	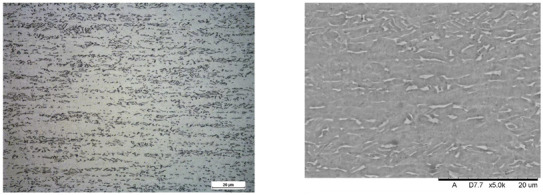	
S2	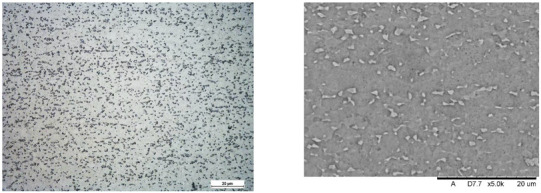	
S3	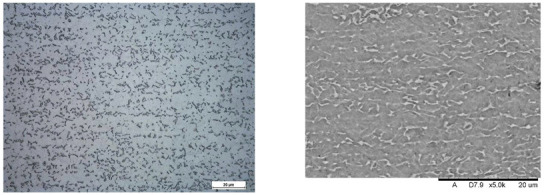	
S4	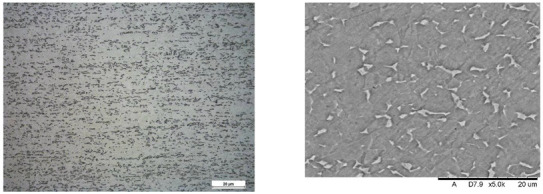	
S5	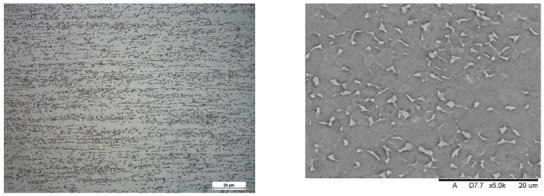	

**Table 9:** EDS phases for S4 sample.

S4-EDS Mark of α Phase	S4-EDS Mark of β Phase
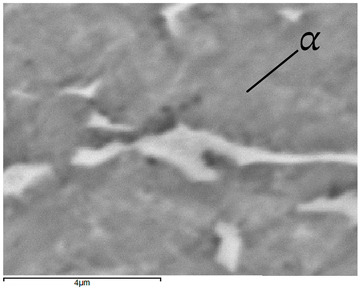	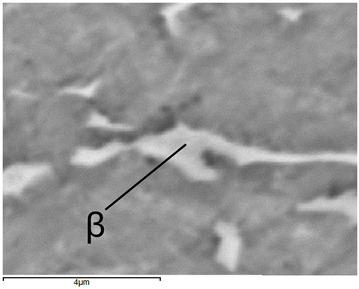

**Table 10:** Chemical composition of α and β phases Ti-6Al-4V alloy in EDS analysis.

Sample	Phase	Amount [% Weight]		
		Ti	Al	V
S1	α	90.9	6.9	2.2
	β	88.1	6.1	5.8
S2	α	91.5	6.5	2.1
	β	85.9	5.9	8.2
S3	α	91.7	7.0	1.3
	β	86.1	6.1	7.8
S4	α	92.3	6.4	1.4
	β	84.0	4.7	11.4
S5	α	92.0	6.1	1.9
	β	81.6	3.8	14.6

**Table 11:** Maximum pull-out force recorded during the experiment and in the numerical analysis.

Sample	$F_{\mathrm{calc}}$	$F_{\exp}$	$\varepsilon_{\%}$
S1	393.36	388.0	−1.36
S2	510.62	523.0	2.42
S3	600.52	237.0	−60.53
S4	666.21	675.0	1.32
S5	609.73	557.0	−8.65

## Data Availability

The original contributions presented in the study are included in the article, further inquiries can be directed to the corresponding authors.
